# The development for emerging biomarkers of lymphangioleiomyomatosis

**DOI:** 10.1186/s13023-024-03455-9

**Published:** 2024-11-29

**Authors:** Liting Huang, Ying Xiao, Lulu Yang, Siying Ren

**Affiliations:** 1 Department of Pulmonary and Critical Care Medicine, The Second Xiangya Hospital, Central South University, Changsha, Hunan 410011 China; 2 Research Unit of Respiratory Disease, Central South University, Changsha, Hunan 410011 China; 3 Clinical Medical Research Center for Pulmonary and Critical Care Medicine in Hunan Province, Changsha, 410011 China; 4 Diagnosis and Treatment Center of Respiratory Disease, Central South University, Changsha, Hunan 410011 China

**Keywords:** Lymphangioleiomyomatosis 淋巴管平滑肌瘤病, Tuberous sclerosis complex结节性硬化症, TSC2, Biomarker 生物标志物, VEGF-D

## Abstract

Lymphangioleiomyomatosis (LAM) is a rare, slowly progressing, low-grade metastatic tumor primarily affecting women. Currently, vascular endothelial growth factor–D (VEGF-D) is the only validated diagnostic biomarker, enabling diagnosis of LAM without the need for lung biopsy in appropriate clinical settings. However, VEGF-D concentrations are normal in about 30% of patients, rendering it insufficient for diagnosing all cases of LAM. There remains a need to identify more non-invasive, safe, sensitive, and specific biomarkers associated with LAM. Therefore, it is imperative to explore novel non-invasive, safe, and specific diagnostic methods for LAM. This article aims to review biomarkers associated with LAM, including potential biomarkers newly discovered or showing advancements in classical biomarkers widely used in LAM, and discuss their application in LAM diagnosis, assessment of disease severity, prediction of treatment response, and prognosis.淋巴管平滑肌瘤病 （LAM） 是一种罕见的、进展缓慢的低级别转移性肿瘤，主要影响女性。目前，血管内皮生长因子-D （VEGF-D） 是唯一经过验证的诊断生物标志物，无需在适当的临床环境中进行肺活检即可诊断 LAM。然而，约 30% 患者的 VEGF-D 浓度正常，不足以诊断所有 LAM 病例。仍然需要确定与 LAM 相关的更多无创、安全、敏感和特异性的生物标志物。因此，当务之急是探索新型无创、安全和特异性的 LAM 诊断方法。本文旨在回顾与 LAM 相关的生物标志物，包括新发现的潜在生物标志物或显示出 LAM 中广泛使用的经典生物标志物的进展，并讨论它们在 LAM 诊断、疾病严重程度评估、治疗反应预测和预后中的应用。

## Introduction 介绍

Lymphangioleiomyomatosis (LAM) is a rare multisystem, low-grade malignant, invasive and metastatic neoplastic disease that mainly occurs in the lungs [1] and is predominant in women of childbearing age, with an incidence of approximately five per million [2]. The pathogenesis of LAM is that deletion of the tuberous sclerosis complex (TSC) genes leads to overactivation of the mammalian target of rapamycin pathway (mTOR) [3], which promotes the proliferation of LAM cells [4]. LAM can arise spontaneously without genetic evidence (S-LAM), or it may be associated with tuberous sclerosis complex (TSC-LAM) which is caused by inactivation mutations in the TSC1 or TSC2 genes [5]. The main characteristic of LAM is diffuse cystic lung destruction caused by the infiltration and proliferation of abnormal smooth muscle-like cells (LAM cells) [6], which can lead to a variety of clinical symptoms such as dyspnea, cough, and recurrent spontaneous pneumothorax [7]. Extrapulmonary symptoms such as chylous effusion, lymphangioleiomyomas and renal angiomyolipomas (AMLs) [2] can also occur.淋巴管平滑肌瘤病 （LAM） 是一种罕见的多系统、低级别恶性、侵袭性和转移性肿瘤性疾病，主要发生在肺部 []，主要发生在育龄妇女中，发病率约为 5/ 万 [2]。LAM 的发病机制是结节性硬化症复合物 （TSC） 基因的缺失导致哺乳动物雷帕霉素通路靶标 （mTOR） 过度激活 []，从而促进 LAM 细胞的增殖 []。LAM 可以在没有遗传证据的情况下自发出现 （S-LAM），也可能与 TSC1 或 TSC2 基因失活突变引起的结节性硬化症 （TSC-LAM） 有关[]。LAM 的主要特征是异常平滑肌样细胞（LAM 细胞）浸润和增殖引起的弥漫性囊性肺破坏 []，可导致呼吸困难、咳嗽和复发性自发性气胸等多种临床症状 []。也可出现乳糜性胸腔积液、淋巴管平滑肌瘤和肾血管平滑肌脂肪瘤 （AML） [] 等肺外症状。

High-resolution CT (HRCT) finding of diffuse thin-walled cysts in the lungs is one of the typical disease features of LAM, but it needs to be combined with clinical manifestations or other findings to confirm the diagnosis of LAM [8]. As the gold standard for the diagnosis of LAM, lung biopsy is difficult to use routinely in clinical practice because of its invasive nature and the risk of bleeding and pneumothorax during operation [9]. Since serum vascular endothelial growth factor–D (VEGF-D) has been used as the only non-invasive diagnostic biomarker for LAM [10], the incidence of lung biopsies in patients with LAM has declined rapidly. But VEGF-D concentrations were normal in about 30% of patients, so it cannot be used to diagnose all patients with LAM.

Therefore, it is necessary to find newer noninvasive, safer and more specific diagnostic methods for LAM. The purpose of this article is to review the biomarkers associated with LAM, including new advances in classical biomarkers widely used in LAM and newly discovered possible biomarkers, and discuss their application in the diagnosis of LAM, evaluation of disease severity, prediction of therapeutic efficacy, and prognosis.

## Vascular endothelial growth factor–D (VEGF-D) — the sole diagnostic biomarker of LAM

VEGF-D is a ligand for the lymphatic growth-factor receptor VEGFR-3/Flt-4 [11], which promotes the remodeling of blood and lymphatic vessels during growth and disease, and plays a key role in tumor metastasis [12]. The abnormal expression of VEGF-D in LAM patients is due to the excessive proliferation of abnormal LAM cells within the lung tissue, which release large amounts of VEGF-D. As LAM cells spread or infiltrate the blood and lymphatic systems in the lung tissue, they stimulate the overproduction of VEGF-D [13, 14].

ATS/JRS guidelines regarded serum VEGF-D as a diagnostic biomarker for LAM patients with HRCT showing typical thin-walled cystic change in the lung, with a diagnostic threshold of 800 pg/ml [10]. The serum VEGF-D concentrations of TSC-LAM patients were slightly higher than that of S-LAM patients [15]. Changes in serum VEGF-D levels appeared to be associated with disease manifestations, including changes in lung function [11], CT scan grade of cysts [16], history of pneumothorax, and the need for oxygen supplementation [17]. The study showed that patients with lymphatic involvement had higher serum VEGF-D levels than patients without lymphatic involvement (*p* = 0.004) [18]. In addition, VEGF-D expression in human lung LAM cells was associated with lung cystic damage (*p* < 0.001) and PFTs (FEV1 and DL_CO_) damage (*p* ≤ 0.004) [19]. Serum VEGF-D concentrations decreased steadily with the duration of sirolimus treatment, and were related to the monthly declines in the values of FEV₁ and FVC after treatment [17, 20]. And there are currently no studies examining the response of VEGF-D in LAM cells to sirolimus treatment.

The serum VEGF-D level was associated with disease progression and is likely a predictor of survival prognosis [21].血清 VEGF-D 水平与疾病进展相关，可能是生存预后的预测指标 []。

## Circulating LAM cells—characteristic tumor cells of LAM

LAM lesions are characterized by multiple pulmonary cysts and LAM nodules [6] consisting mainly of central LAM cells and surrounding stromal cells [7]. LAM cells with *TSC2* mutations and loss of heterozygous (LOH) deletions [22] were characterized by abnormal morphology, excessive proliferation, irregular migration and reduced autophagy [18].

LAM cells could be detected in diseased lung tissues [23] or various body fluids, suggesting that LAM might be a genetic disease involving multiple organs [2]. Crook et al. [23] isolated LAM cells with TSC2 LOH from the blood of 55% of LAM patients (*n* = 60) using density gradient centrifugation. Cells with TSC2 LOH were found in the urine from 11 of 14 AML patients (79%), and in the chylous fluid from 1 of 3 LAM patients (33%). In addition, LAM cells were also detected in the body fluid of 1 patient after double lung transplantation. However, there was no correlation between the detection of LAM cells in the body fluids of LAM patients and the severity of their lung lesions. Loss of LAM cells with TSC2 LOH in body fluids after rapamycin treatment in LAM patients was associated with duration of treatment and premenopausal status [24]. Cai et al. [24] also found that LAM cells with TSC2 LOH were detected in 100% of blood specimens and 75% of urine specimens before rapamycin treatment in LAM patients. During a mean of 2.2 ± 0.4 years of sirolimus treatment, the detection rate of LAM cells decreased significantly to 25% in blood (*p* < 0.001) and 8% in urine (*p* = 0.003).

Typical LAM cells and positive immunohistochemical staining of human melanoma black 45(HMB-45) antibody in lung biopsy are important methods for the diagnosis of LAM [2]. Circulating LAM cells are also possible biomarkers for diagnosing LAM and predicting the efficacy of sirolimus. However, in other pulmonary cystic diseases, TSC2-deficient cells can also be detected in peripheral blood and urine [25]. Therefore, the presence of TSC2-deficient cells cannot be used to diagnose LAM, further evaluation based on the patient’s clinical presentation and high-resolution computed tomography (HRCT) is required for diagnosis [26] (Table 1).

**Table 1:** Summary of each type of biomarker in this review

Types	Biomarkers	Detection sites	Positive rate in serum/plasma(%)	Drugs	References
Sole diagnostic biomarker	VEGF-D	Serum	82	Rapamycin	[108]
Characteristic tumor cells特征性肿瘤细胞	Circulating LAM cells	Blood; urine; chylous fluid; LAM nodules LAM 结节	100		[25]
Proteins with cystic lung destruction具有囊性肺破坏的蛋白质	MMPs MMP	Serum; 血清; blood; 血; urine; 尿; BALF; 巴尔夫; LAM cells; LAM 细胞; LAM nodules	54(MMP-2) 54（MMP-2）	Doxycycline 多西环素	[30]
	GPNMB	Serum; LAM nodules			[44]
	CATK	LAM nodules			[47]
	VDBP	Serum			[52]
	Moesin	Serum	81		[54]
	FGF23	Serum	59.46		[56]
Biomarkers associated with tumor immune microenvironment	PD-1/PD-L1	LAM nodules		PD-1/PD-L1 inhibitors	[60–62]
	NKG2D/NKG2DLs	NK cells in the blood and lung tissues of LAM patients			[71]
	GD3	Serum; LAM nodules			[60]
	Chemokines and their receptors	BALF; LAM cells; LAM nodules			[78–81]
	NE	LAM nodules			[45]
Other biomarkers	MicroRNAs	Serum; LAM cells; LAM nodules			[90–92]
	Lipids	Serum; Blood; LAM cells; LAM nodules		Aspirin and other COX, PLA2 or EP3 inhibitors	[93–98]
	Amino acids	Blood	73.8(methionine) 95.1(cetic acid)		[100]
	Serum proteins	Serum			[101]
	CA-125	Serum; LAM nodules	24.9		[102]
	Endostatin	Serum	82		[104]
	sKL-6	Serum			[105]
	Soluble immune-related proteins	Serum			[107]

VEGF-D: vascular endothelial growth factor–D; MMPs: Matrix metalloproteinases; GPNMB: Glycoprotein non-metastatic melanoma protein B; CATK: Cathepsin K; VDBP: Vitamin D binding protein; FGF23: Fibroblast growth factor; PD-1/PD-L1: Programmed cell death protein 1/ PD-ligand 1; NKG2D/NKG2DLs: Natural Killer Group 2 member D/NKG2D ligands; GD3: Ganglioside D3; NE: Neutrophil elastase; CA-125: Cancer antigen 125; Skl-6: Serum Krebs von den Lungen-6

## Proteins with cystic lung destruction in LAM

The occurrence of pulmonary cystic damage in LAM could be associated with the abnormal expression of some proteases with lung injury function and lung protective proteins. These proteins might be used as potential biomarkers to monitor the severity of LAM.

### Matrix metalloproteinases (MMPs)

MMPs are a group of proteases that degrade the extracellular matrix and reshape connective tissue, promoting tumor growth, invasion and metastasis [27]. The pathogenesis of pulmonary cystic damage in LAM might be the expression imbalance between MMPs and their tissue inhibitors (TIMPs) [28].

The MMP-2 [29] and MMP-9 [6] staining on the surface of LAM cells were positive, and the MMP-2 [30] and − 9 [31] in the serum of LAM patients were significantly increased, suggesting that they might have diagnostic significance for LAM. Tissue inhibitors of MMP-3 could inhibit the proteolytic effect of some MMPs [32], and the expression levels in LAM lung parenchyma were lower than that in normal lung tissue. Nascimento et al.) [6] found that high expression of MMP-9 in human LAM cells was correlated with decreased cyst density, nodular density (*p* < 0.001) and PFT (FEV1 [32]and DLCO) (*p* < 0.001). Chang et al. [18] also found that elevated serum MMP-2 levels were associated with improved lung function (FEV1 and DL_CO_) (*p* = 0.0023). However, the study of Baldi et al. [33] showed that HRCT showed no correlation between the degree of pulmonary cystic damage and serum MMP-2 and − 9. MMP-2, -3, and − 9 expressions increased in bronchoalveolar lavage fluid (BALF) of TSC2-deficient mice compared with control mice (*p* < 0.001) [34], which was associated with lung destruction caused by TSC2-deficient mice. Most of the results supported that the high expression of MMPs could be significantly related to the pulmonary cystic progression of LAM, and detection of MMPs in blood or BALF may have significant value in predicting the disease progression of LAM.

Doxycycline is an antibiotic that inhibits the activity of several matrix metalloproteinases, including MMP-2 [18] and − 9 [35]. After treatment with doxycycline and other drugs, the expression of various MMPs decreased, indicating that MMPs can be used as a possible biomarker to predict the efficacy of LAM. Urinary MMP-9 (from 10,487 pg/mL baseline to 4,061 pg/mL; *p* < 0.001) levels in patients with LAM after doxycycline treatment were significantly decreased [36]. However, Chang et al. [37] performed a two-year randomized, double-blind trial, comparing the matrix metalloproteinases inhibitor doxycycline with placebo on the progression of LAM, found doxycycline had no effect upon vital capacity, gas transfer, shuttle walk distance or quality of life. Urine matrix metalloproteinases-9 measurements were lower with doxycycline treatment (*p* = 0.03). This finding was significant to the extent that the ATS guidelines do not recommend the use of doxycycline for LAM [10]. Moir et al. [38] revealed that doxycycline decreased the total secretion of MMP-2 and the expression of active MMP-2 on LAM cells (*n* = 5; *p* < 0.05). Lee et al. [39] found that rapamycin did not reduce MMP-2 secretion in TSC2-deficient cells, suggesting that the increase in MMP-2 levels appeared to be independent of mTORC1. Rapamycin and simvastatin had different inhibitory effects on MMP-2,3 and 9 in BALF of TSC2 deficient mice (*p* < 0.001) [34]. This result cannot exclude the association between abnormal MMP-2 expression and mTORC1, and further studies are needed. In addition, LAM patients with higher MMP-9 expression level had a lower survival rate and a higher risk of receiving lung transplantation (*p* < 0.03) [6], indicating that the monitoring of MMP-9 can be used to assess the prognosis of LAM.

Thus, the level changes of MMPs in different fluids could be significant indicators of disease progression and prognosis in LAM patients. More therapies associated with MMPs and TIMPs are possibly effective for LAM patients.

### Glycoprotein non-metastatic melanoma protein B (GPNMB)

GPNMB is an intracellular transmembrane protein that regulates physiological processes in normal tissues [40]. GPNMB was overexpressed in the extracellular domain (ECD) of cancer cells of many malignancies, including melanoma, glioblastoma, and triple-negative breast cancer, and has been shown to be associated with tumor migration, invasion, and metastasis [41]. GPNMB was shed from the cell surface and released into the microenvironment through MMP cleavage [42]. The exfoliated ECD interacting with integrins could promote the expression and secretion of MMP and cytokines in LAM patients [40].

Prizant et al. [43] identified positive GPNMB staining in LAM nodules in lung, uterus and lymph nodes of LAM patients. Taya et al. [40] detected that the ECD of GPNMB fell off from the cell surface of TSC2-deficient cells and entered the circulation, and the soluble GPNMB content in serum of LAM patients was significantly higher than that of healthy control samples, suggesting that GPNMB could be used as a new biomarker for the diagnosis of LAM. In addition, Gibbons et al. [44] demonstrated that GPNMB’s ectodomain was present at higher levels in LAM patient serum compared to healthy controls, and that ectodomain levels decrease with mTORC1 inhibition. Moreover, Taya et al. [45] found that soluble GPNMB recruited angiogenic factors such as MMP-2 and MMP-9 into the tumor microenvironment, promoting tumor migration and invasion. SiRNA-mediated downregulation of GPNMB expression led to decreased expression of MMP-2/9, which verified the relationship between GPNMB and MMPs. Therefore, detecting the expression of GPNMB and soluble GPNMB in LAM nodules has certain potential value for the diagnosis of LAM. Based on the tumor promoting effect of GPNMB and its localization on the surface of tumor cells, targeting GPNMB and its mRNA as therapeutic intervention may inhibit the pulmonary cystic damage and tumor growth of LAM, and slow the progression of the disease.

### Cathepsin K (CATK)

CATK is a papain-like cysteine protease that had high stromal degradation activity for collagen and elastin [46] and was expressed on osteoclasts and tumor stromal fibroblasts, but not in body fluids or normal lung tissue [47]. Activation of CATK required acidification of the extracellular environment in tumor stroma [47]. CATK destroyed lung structure and reshaped lung parenchyma after activation [48], which might be related to the formation of LAM lung cysts [49].

Dongre et al. [47] found that CATK was increased 40-fold in LAM nodules than in normal lung tissue (mean 0.375 vs. 0.00092; *p* < 0.0001), expressed on the surface of LAM lung fibroblasts. Rapamycin (10 nmol/L) reduced CATK gene expression in LAM lung tissue by inhibiting extracellular pH induced by TSC2-/- cells. This suggests that the expression or activation of CATK may depend on mTORC1. Miller et al. [49] showed that the number of fibroblasts in LAM nodules gradually exceeded LAM cells as the disease progressed.

Meanwhile, increased CATK expression in LAM nodular tissue was associated with decreased FEV1 (95%CI -1.11 to -0.18) and DLco (95%CI -0.96 to -0.05) [49], suggesting that the aggregation of LAM fibroblasts led to CATK overexpression. It could promote a progressive decline in lung function with LAM. CATK in nodules of LAM might be an effective biomarker for the diagnosis and disease progression of LAM, but its detection is challenging. More studies are needed to explore whether CATK is abnormally expressed in the blood or other fluids with LAM patients (Table 2).

**Table 2:** Clinical trial table for biomarker-targeted drugs

Drugs	Target	Trial phase	Trial status	Key findings	References
Rapamycin	VEGF-D; m-TOR	II	Completed	Despite the occurrence of numerous adverse events during long-term sirolimus treatment in Asian patients with LAM, the patients’ FEV1, FVC, and quality of life (QOL) parameters remained stable	[109]
Doxycycline	MMPs	II	Completed	Doxycycline had no effect on vital capacity, gas transfer, shuttle walk distance, or quality of life. Urine matrix metalloproteinases-9 measurements were lower in the doxycycline treatment group (*p* = 0.03)	[37]
Celecoxib	COX-2	I	Completed	The trial has confirmed the safety of daily oral administration of 200 mg celecoxib for patients with LAM. Larger-scale Phase II/III trials are needed to determine the efficacy of this treatment, particularly in subsets of patients with LAM and high VEGF-D levels	[99]

### Vitamin D binding protein (VDBP)

VDBP, also known as group-specific component globulin (GC-globulin), is mainly produced by the liver and is the main transporter of vitamin D in the blood metabolism process [50]. VDBP could potentially protect lung injury in LAM by increasing the accessibility of vitamin D in the lungs and then inhibiting the activation of MMP-2 and − 9 in lung fibroblasts [51].

Miller et al. [52] discovered that VDBP was associated with disease severity and prognosis in LAM patients. Proteomic analysis showed that the serum VDBP levels of LAM patients were 2.6 times lower than that of healthy controls. VDBP concentrations were consistently lower in patients with progressive disease (> 50 mL FEV1 loss per year) than in patients with stable lung LAM (< 50 mL FEV1 loss per year) (221 ± 89 µg· ml-1 vs. 299 ± 90 µg· ml-1 stability; *p* = 0.001). Serum VDBP levels were positively correlated with lung DLco (*p* = 0.01), but not with FEV1 and FVC. Serum VDBP levels might be an individual marker of disease development, independent of age, menopausal status, nature of symptoms, tuberous sclerosis, angiomyolipoma, lymphatic disease, or serum VEGF-D levels. In addition, a single nucleotide polymorphism (SNP) in VDBP gene GC was associated with time to death or lung transplantation [51, 52]. CC genotype RS4588 had the shortest median time to death or transplantation of all VDBP genotypes (*p* = 0.014). Serum VDBP may serve as a biomarker to predict the severity of the disease in LAM patients, and the SNP in its haplotype is considered as a possible genetic marker associated with disease progression.

### Moesin

Moesin is a member of the ERM (Ezrin/Radixin/Moesin) family of proteins, which is a connecting protein between the cell membrane and the cytoskeleton, and is capable of stabilizing the cell membrane structure [53]. Moesin was found negatively correlated with FEV1%pred, FEV1/FVC, and DLCO%pred (*p* = 0.0181, *r* = − 0.3398; *p* = 0.0067, *r* = − 0.3863; *p* = 0.0010, *r* = − 0.4744) in lung tissues and serum of the patients with LAM. A composite score combining moesin and VEGF-D improved prediction for sirolimus treatment, compared with each biomarker alone. Moreover, elevated levels of moesin were related to lymphatic involvement in patients with LAM [54]. Therefore, higher levels of moesin in serum may indicate impaired lung function and lymphatic involvement in patients with LAM, suggest a more serious condition, and provide clinical guidance for sirolimus treatment.

### Fibroblast growth factor (FGF23)

FGF23 is a hormone that regulates blood phosphorus and is distributed throughout the body [55]. Esposito et al. [56] found serum FGF23 levels were higher in the LAM group than in the control group. And lower FGF23 levels were associated with impaired DLCO (*p* = 0.04), particularly for those with isolated diffusion impairment with no other spirometric abnormalities (*p* = 0.04). FGF23 alone or in combination with other molecules needs to be validated as a biomarker of LAM activity in future clinical research.

## Biomarkers associated with tumor immune microenvironment of LAM

LAM cells consist of two subgroups: (1) small spindle-shaped cells expressing smooth muscle-specific proteins, mainly α -smooth muscle actin; (2) epithelioid-like cells that expressed markers of melanoma cells and immature melanocytes, such as ganglioside D3 [57], gp100, and MelanA/ MART-1 [58], which could be recognized by T cells [59]. Klarquist et al. [60] found that LAM cells cultured were susceptible to melanoma reactive cytotoxic T cells in vitro. Compared with healthy lung tissue, the number of T cells in LAM lung tissue was only slightly and insignificantly increased, but immune checkpoint molecules in LAM nodules were overexpressed [60–62], indicating the evidence of T cell depletion in the tumor microenvironment of LAM. Treatment with immune checkpoint inhibitors might be new therapeutic methods for eradicating LAM tumor cells. In addition, other targets have been found in the immune microenvironment of LAM, which provided a basis strategy for the exploration of anti-tumor immunotherapy for LAM.

### Programmed cell death protein 1(PD-1) and PD-ligand 1(PD-L1)

PD-1, predominantly expressed on tumor-infiltrating T cells, is an important immune checkpoint receptor that enabled tumors to evade immune surveillance [63]. After ligating with overexpressed PD-L1 in the tumor microenvironments, such as tumor cells, stromal cells, and/or antigen-presenting cells, PD-1 transmitted inhibitory signals to T cells, leading to programmed death and the exhaustion of T cell [64, 65]. Targeting PD-1/PD-L1 has proven to be effective immune checkpoint blockade therapies to stimulate antitumor immunity in a variety of cancers [66].

PD-1 was highly expressed on T cells infiltrating in LAM nodules [60–62]. The study of Liu found that PD-L1 was highly expressed on hematopoietic cells, but there was no significant difference in the expression on the surface of tumor-infiltrating macrophages and monocytes in LAM nodules compared with healthy lung tissue [61]. In contrast, Maisel found that PD-L1 was highly expressed on stromal cells and antigen-presenting cells (APCs) in human LAM lung tissues and TSC2^−^ lesions in LAM mouse models [62].

Anti-PD-1 therapy enhanced T cells infiltration in TSC2-deficient tumors, inhibited tumor growth by 62% (*p* < 0.0001) [61] and improved the survival rates of LAM mice (*p* < 0.0001) [62]. Meanwhile the study of Liu found that anti-PD-1 blockade after sequential rapamycin therapy slowed the growth rates of tumor cells in TSC2-deficient tumors [61]. And Pluvy et al. [67] reported a case of using nivolumab (a PD-1 blocking antibody) combined with sirolimus for treating metastatic lung adenocarcinoma in a patient with sporadic LAM. The treatment showed good safety and tolerability without affecting the efficacy of nivolumab. Therefore, overexpressed PD-1/PD-L1 in LAM nodules might be molecular targets for checkpoint blockade immunotherapy in LAM patients, and PD-1/PD-L1 blockade monotherapy or combination therapy of rapamycin and PD-1/PD-L1 inhibitors might be promising approaches for LAM. However, if such a treatment option is considered for LAM patients, close monitoring is essential to ensure there are no LAM exacerbations or any toxic effects.

### Natural killer Group 2 member D (NKG2D) and NKG2D ligands (NKG2DLs)

NKG2D is normally expressed on many cytotoxic immune cells including NK cells as an activating immune receptor [68]. Its ligands, NKG2DLs, are expressed in many tumor tissues, but not in normal tissues [69]. The combination of NKG2D expressed by NK cells and NKG2DLs on the surface of tumor cells improved NK cell cytotoxicity against tumor cells and played an immune surveillance role. Shedding of NKG2DLs from tumor cells increased the concentrations of its immune soluble form (sNKG2DLs) [68], leading to down-regulation of NKG2D expression and inhibition of NK cell function, which is a mechanism of tumor immune escape [70].

Osterburg et al. [71] found that NKG2DLs (ULBP3 and/or ULBP2) were detected in human lung LAM cells, but not in normal lung tissues surrounding LAM lesions and soluble ULBP2(mean = 575 pg/ml ± 142) and ULBP3(mean = 8300 pg/ml ± 1515) were increased in serum of LAM patients. Compared with healthy controls, NKG2D positive percentage of CD3^–^CD56^bright^CD16– and CD3^–^CD56^dim^CD16^+^ NK cells and the number of circulating NK cells in patients with LAM were lower compared with healthy controls (*n* = 7; *p* < 0.05). The shedding of NKG2DLs and the change of NK cell population suggested that LAM patients might have decreased NK cell cytotoxicity and surveillance functions against tumors. Subsequently, Foot et al. [72] discovered that NKG2DLs was expressed in LAM cells of TSC2-/- angiomyolipoma and TSC2-/- mouse embryonic fibroblasts, which was consistent with the above findings. Patients expressing ULBP2 and ULBP3 had a lower rate of FEV1 decline over 48 months (-124 ± 30 mL/year) than patients without serum sNKG2DLs (-32.7 ± 10 mL/year) (*p* < 0.05) [71], which meant the presence of sNKG2DLs might be related to the pulmonary cystic destruction in LAM patients.

Therefore, NKG2DLs is a promising biomarker and new therapeutic target associated with disease progression in LAM patients, but the mechanisms of functional alterations in NK cells and sNKG2DLs abnormal expression remain unclear. Borchers et al. [73] showed that NK cells in the blood and lung tissues of LAM patients were highly reactive, which could be related to the stimulation of VEGF receptor signaling pathway. The relationship between NK cells and LAM needs further study.

### Ganglioside D3(GD3)

GD3 is the only tumor-associated glucolipid expressed on melanoma and neuroendocrine tumor cells [74] and is associated with the proliferation and invasion of melanoma cells [75]. GD3 could be cross-presented by CD1d expressed on APCs or tumor cells and recognized by invariant natural killer T (iNKT) cells [76].

Gilbert et al. [57] found that CD1d and GD3 were found co-expressed in LAM tissues probably associated with NKT-cell-mediated cytotoxicity, however the numbers of infiltrating NKT cells in LAM lungs had no difference with normal lung tissues. Developing vaccines targeting GD3 to enhance NKT-cell infiltration may further improve antitumor responses. GD3 expression was twofold higher in LAM lung tissue than in healthy control lung (*p* = 0.03), while serum GD3 antibodies were significantly threefold lower in LAM patients compared with normal samples (*p* = 0.005). It demonstrated that the levels of GD3 or its serum antibodies could be biomarkers of diagnosis in LAM. Meanwhile LAM cells were targeted by complement mediated cytotoxicity with antibodies to GD3 which could become a new target of antibody treatment with LAM patients. Thus, GD3 is possible to be a suitable target for immunotherapy of LAM.

### Chemokines and their receptors

Chemokines (C, CC, CXC, CX3C) is a large family of cytokines with chemotactic activity. Their receptors can be expressed in different cancer cells and promote the growth and survival of neoplastic cells [77]. The interactions of chemokines and chemokine receptors in tumor induces the infiltration of different immune cells like macrophages of the tumor microenvironment.

Cui et al. [78] found the overexpression of CCL2/MCP1 in TSC2-deficient cells due to activation of mTORC1 and Syk signaling. Pacheco-Rodriguez et al. [79] also found that concentrations of CCL2, CXCL1, and CXCL5 were significantly higher in BALF from LAM patients than from healthy volunteers. Meanwhile, receptors CXCR1, CXCR2, CXCR4, CCR2, CCR7, and CCR10 were present more frequently than other receptors in LAM cells within lung nodules. Klarquist et al. [80] discovered that the expression of macrophages in LAM lung tissue was fivefold greater than that of normal lung tissue, and Maisel et al. [62] also found that macrophages were increased in Tsc2-null lung lesions from a metastatic model of LAM (*p*<0.001). The study of Atochina-Vasserman et al. [81] demonstrated that macrophages and neutrophils were recruited into lung lesions which was associated with the upregulation of CCL2/MCP1 and CXCL1/KC chemokines in BALF fluids with TSC2^−^ lesions. Pacheco-Rodriguez et al. [79] also showed that LAM cells expressed CCR2 could be attracted by CCL2 and appeared to metastasis which reflected the potential effect of CCL2 in recruiting LAM cells into lung.

The upregulation of chemokines and their receptors and the recruitment of macrophages may be involved in tumor microenvironment of LAM. But the category and function of macrophages in LAM tissues is unknown currently. Various elevated chemokines and their receptors could be biomarkers for evaluating immunotherapy effect in LAM and CCL2-CCR2 signaling could be promote the migration of LAM cells and influence tumor progression.

### Neutrophil elastase (NE)

NE is a serine protease secreted by varieties of immune cells including neutrophils [82] and circulating myeloid-derived suppressor cells (MDSCs) [83]. MDSCs are a heterogeneous population of activated immature myeloid cells associated with tumor growth [84]. MDSCs and NE are overexpressed in tumor microenvironment when MDSCs are not expressed in the blood under normal physiological conditions [85].

Gibbons et al. [40] showed that MDSCs specially granulocytic myeloid cells (G-MDSCs) were found presented in uterus (*p* < 0.05), lung and peripheral blood (*p* < 0.01) of Tsc2-null mice and infiltrated selectively in human LAM lung tissue [45]. The infiltration of MDSC in the LAM tumors might be connected with CXCL5/CXCR2 interactions and could promote tumor progression [45]. NE, likely expressed by MDSC, was also highly expressed in myometrial LAM-like Tsc2-null mice tumors [43] and promoted the proliferation migration and invasion of Tsc2-null cells [45]. And the overexpression of NE might induce cystic lung destruction with degrading elastin [45]. Therefore, NE or MDSC could be therapy targets to suppress tumor growth and migration in LAM.

## Other biomarkers that could be detected in serum of LAM

### MicroRNAs (miRNAs or miRs)

MiRNAs are a group of non-coding single-stranded short RNA that regulate gene expression mainly by affecting the stability of transcripts [86]. MiRs were involved in the pathogenesis of many malignancies and several lung diseases, including asthma, pulmonary hypertension, and idiopathic pulmonary fibrosis [86]. The abnormal expression of a variety of miRs has been proved to have different effects on the occurrence and development of tumors [87], which might be related to the complex mTOR signal network of LAM.

Many miRs expressed abnormally in patients with LAM might serve as biomarkers for the diagnosis of LAM, and some miRs could become targets for molecular therapy of LAM. It was found that the expression of miR-25 increased in LAM cells of patients. In LAM cells, TSC1 was the target of miR-25-mediated gene silencing [88], which meant that targeting miR-25 has potential as a new molecular therapy for LAM. MiR-124-3p was down-regulated in LAM tissues of patients (*n* = 4; *p* < 0.05) and inhibited the apoptosis of TSC2-deficient cells through the oxidation of peroxisomal fatty acids mediated by retinoid X receptor α (RXRα) [89], indicating that RXRα might be the target of miR-124 in LAM. Takimoto et al. [90] found that all miRs from serum extracellular vesicles (EVs) in LAM patients were changed, with 26 miRs increased by more than two times and three miRs reduced by more than half compared with healthy controls.

Some miRs, named as “RapamiRs”, were abnormally expressed after treatment with rapamycin [86] and could serve as possible biomarkers to monitor the efficacy of rapamycin. In the LAM cell model, miRs-29b, 21, 24, 221, 106a, 199a and other miRs expressions were up-regulated after treatment with rapamycin (*p* < 0.05), and miR-21 expression was the most significantly up-regulated (*p* < 0.01). MiR-29b was up-regulated after treatment in TSC2-deficient cells, but it also promoted TSC2-deficient cell growth by rendering the overactivation of mTORC1 as an oncomiR (*p* < 0.001) [91]. MiR-29b inhibitors in combination with rapamycin further inhibited tumor-related cellular processes compared with rapamycin alone. These data suggested that the abnormal expression of RapamiRs was mTOR dependent.

Rossi et al. [92] analyzed the serum miRNA expression profiles of 27 LAM patients and 33 healthy subjects and found that miR-1972 and miR-186-5p are potential serum biomarkers for LAM, particularly when combined with miR-320 family members, especially in cases where other clinical standards, such as VEGF-D concentration, are insufficient. These data suggest that combinations of miRNAs may improve LAM diagnosis. Future studies could test the ability of these miRNAs to distinguish LAM from other confounding pulmonary diseases, as well as their prognostic value and ability to predict treatment response.

### Lipids

Lipids metabolism of LAM patients were found altered according to the studies of Priolo et al. [93] and Bottolo et al. [94]. Priolo et al. [93] revealed that fifteen lipid species were overexpressed in plasma of LAM patients (*p*<0.05), including nine triacylglycerol, two phosphatidylcholines, and four lysophosphatidylcholines (LPC) species (C16:0, C18:0, C18:1, and C20:4), and also 43 lipid species were abnormal in the TSC2^−^ cells (*p*<0.05) compared with TSC2^+^ cells. A large cohort of LAM patients from Bottolo et al. [94] showed that the metabolism of sphingolipid, fatty acid and phospholipid were associated with the rate of decrease of FEV1 and total disease burden of LAM (*p*< 0.05). There were differences of glycerophospholipid metabolism between those TSC-LAM but not S-LAM patients who took rapamycin treatment and who did not. Besides, Gu et al. [95] collected blood samples for nuclear magnetic resonance (NMR) testing and found methionine and acetic acid in patients with LAM had the highest diagnostic efficiency, and that methionine was significantly associated with pneumothorax (*p* < 0.05) and creatinine with uterine fibroids (*p* < 0.05). In addition, acetone and creatinine are promising metabolic markers to differentiate S-LAM from TSC-LAM.

Li et al. [96–98] discovered that the expression of cyclooxygenase-2 (COX-2) and adipocyte-specific phospholipase A2 (AdPLA2, also called PLA2G16), which are associated with prostaglandin biosynthesis, were increased in TSC^−^ cells in vitro and in vivo with in a rapamycin-insensitive manner. COX-2 was overexpressed in lung nodules of LAM patients compared with in control lungs(*n* = 3; *p*<0.05). AdPLA2 was upregulated in smooth muscle-like cells of LAM lung nodule (*n* = 6; *p*<0.01). Then, the levels of prostaglandin E2 (PGE2) [96] and its cognate receptor, EP3 protein [98] were also discovered increased in the serum of LAM patients than healthy controls (*p* < 0.01). And it was found that PGE2 was correlated positively with VEGF-D (*p* < 0.05) in LAM subjects [96]. These studies suggested that serum PGE2 could be a biomarker of disease progression of LAM.

Abnormal phospholipid metabolism in addition to mTORC1 pathway could promote the exploration of other disease pathogenesis and novel treatment in LAM. Aspirin and other COX, PLA2 or EP3 inhibitors [96–98] may be novel treatments of LAM patients due to the ability of suppressing the growth of TSC2^−^ cells. El-Chemaly et al. [99] demonstrated the possible therapeutic effect and safety of celecoxib, a COX-2 inhibitor, for LAM patients in the early stage or with elevated VEGF-D levels.

### Amino acids

Gu et al. [100] collected blood samples for NMR testing and found methionine and acetic acid in patients with LAM had the highest diagnostic efficiency, and that methionine was significantly associated with pneumothorax (*p* < 0.05) and creatinine with uterine fibroids (*p* < 0.05). In addition, acetone and creatinine were promising metabolic markers to differentiate S-LAM from TSC-LAM.

### Serum proteins

Lamattina et al. [101] measured 279 serum proteins of samples in the SAIL trial and discovered that the acetyl-CoA carboxylase complex and coagulation factor II could play important roles in LAM pathogenesis, which was involved in wound healing, blood homeostasis, and inflammation. And some possible predictors of lung function such as VEGFR-3, CCL21, TFF3 and CD5L/AIM also were discovered over the treatment period.

### Cancer antigen 125 (CA-125)

CA-125 was expressed in human LAM lung nodules, and serum CA-125 levels were above normal in only 24.9% of 241 LAM patients (> 34 U/mL) [102]. Higher serum CA-125 levels were associated with pleural effusions (*n* = 60; *p*<0.01) and reduced pulmonary function (FEV1: *p* < 0.001; DL_CO_: *p* < 0.001), but some patients with elevated CA-125 level had no history of pleural effusions. Thus, the source of CA-125 could be LAM nodule not pleura or pleural effusions. Levels of serum CA-125 in patients were decreased after sirolimus treatment (*p*<0.002).

CA-125 is a tumor-associated antigen as a part of the mucin 16 (MUC16) [103]. MUC16 was expressed on the epithelial surface lining of multiple organs and could be released by proteolytic cleavage with NE, MMP-7, and MMP-9 [103]. The elevated serum CA-125 levels could be associated with the high expression of these proteinases in LAM. Thus, CA-125 may be regarded as a biomarker for diagnosis, disease progression and treatment in LAM. The pathogenesis about elevated CA-125 should be explored further.

### Endostatin

Lamattina et al. [104] showed that serum endostatin levels were associated with DL_CO_ impairment of LAM patients and TSC-LAM but not S-LAM. In comparison to S-LAM patients, TSC-LAM patients (54.2 ± 6.1; *n* = 16) had higher serum endostatin levels which reflected the difference of the disease pathogenesis with two types.

### Serum Krebs von den Lungen-6 (sKL-6)

D’Alessandro et al. [105] found that the sKL-6 levels of LAM patients were highly expressed compared with which of healthy controls (*p* = 0.0133) with the cut-off value of 250.5 U/ml. SKL-6 levels with LAM were negatively correlated with lung function including FEV1 and FVC (*p* = 0.042). And the sKL-6 levels in IPF patients were higher than that in LAM patients (*p* = 0.0002) and in healthy group with the best cut-off value of which was 1124 U/ml. The result was consistent with the view that sKL-6 could be a potential biomarker with various ILD [106].

### Soluble immune-related proteins

Liu et al. [107] collected serum samples from 67 LAM patients and 49 healthy controls, comparing 59 serum immune factors measured by the Luminex method. They found that LAG-3 may have better predictive value than VEGF-D and showed significant differences between patients without elevated VEGF-D and healthy individuals. Additionally, IL-18 was positively correlated with lung function and 6-minute walk test distance, and negatively correlated with St. George’s Respiratory Questionnaire scores and pulmonary artery systolic pressure, indicating its association with disease severity. Therefore, LAG-3 and IL-18 could serve as biomarkers for diagnosis and disease progression prediction.

## Challenges and future perspectives

Developing biomarkers for tumor immunotherapy can offer crucial guidance for precision medicine in patients with LAM. As a rare disease with a still largely unexplored pathogenesis, there is a pressing need for highly sensitive and specific biomarkers to enhance clinical care and deepen our understanding of LAM. While VEGF-D currently serves as the only validated diagnostic biomarker in appropriate clinical contexts, obviating the need for lung biopsy to diagnose LAM, additional biomarkers are actively being researched by numerous investigators, considering the diverse symptoms and underlying pathogenesis of LAM. Especially in some patients with no significant increase in VEGF-D concentration, other biomarkers, such as ARN, need to be tested. Moreover, a few biomarkers are only detected in LAM cells and LAM nodules, and further research is required to determine if they can be detected in blood or serum. In the future, we can significantly improve patient care by developing more precise LAM treatments through biomarkers and facilitating simplified clinical trials. This will provide patients with personalized treatment, enhancing their quality of life and survival.

## Data Availability

Data sharing is not applicable to this article as no datasets were generated or analysed during the current study.

## Abbreviations

LAM: Lymphangioleiomyomatosis
TSC: Tuberous sclerosis complex结节性硬化症
AMLs 反洗钱: Angiomyolipomas
HRCT HRCT （HRCT） 检测: High-resolution CT 高分辨率 CT
VEGF-D VEGF-D 型: Vascular endothelial growth factor–D血管内皮生长因子–D
LOH 奥运会: Loss of heterozygous 杂合子缺失
HMB-45 HMB-45 型: Human melanoma black 45 人黑色素瘤 black 45
MMPs: Matrix metalloproteinases
GPNMB: Glycoprotein non-metastatic melanoma protein B
ECD: Extracellular domain
CATK 卡特: Cathepsin K 组织蛋白酶 K
VDBP: Vitamin D binding protein维生素 D 结合蛋白
SNP SNP （SNP）: Single nucleotide polymorphism单核苷酸多态性
ERM 企业风险管理: Ezrin/Radixin/Moesin
FGF23: Fibroblast growth factor 成纤维细胞生长因子
PD-1/PD-L1 PD-1/PD-L1 型: Programmed cell death protein 1/PD-ligand 1程序性细胞死亡蛋白 1/PD 配体 1
APCs APC: Antigen-presenting cells
NKG2D/NKG2DLs NKG2D/NKG2DL: Natural Killer Group 2 member D/NKG2D ligands自然杀伤细胞第 2 组成员 D/NKG2D 配体
APCs APC: Antigen-presenting cells 抗原呈递细胞
GD3: Ganglioside D3 神经节苷脂 D3
iNKT 墨: Invariant natural killer T不变的自然杀伤剂 T
NE 不: Neutrophil elastase 中性粒细胞弹性蛋白酶
MDSCs MDSC: Myeloid-derived suppressor cells髓源性抑制细胞
G-MDSCs: Granulocytic myeloid cells
miRNAs/miRs: MicroRNAs 微小RNA
RXRα RXRa: Retinoid X receptor α 类 A A X 受体 α
EVs 电动汽车: Extracellular vesicles 细胞外囊泡
LPC LPC 公司: Lysophosphatidylcholines 溶血磷脂酰胆碱
NMR 核磁共振: Nuclear magnetic resonance核磁共振
COX-2: Cyclooxygenase-2 环氧合酶-2
AdPLA2/PLA2G16: Adipocyte-specific phospholipase A2
PGE2: Prostaglandin E2
CA-125: Cancer antigen 125
MUC16: Mucin 16 粘蛋白 16
sKL-6: Serum Krebs von den Lungen-6 (sKL-6)血清肺癌 6 （sKL-6）
